# The global research of microbiota in colorectal cancer screening: a bibliometric and visualization analysis

**DOI:** 10.3389/fonc.2023.1169369

**Published:** 2023-05-05

**Authors:** Junhai Zhen, Chuan Liu, Fei Liao, Jixiang Zhang, Huabing Xie, Cheng Tan, Weiguo Dong

**Affiliations:** ^1^ Department of General Practice, Renmin Hospital of Wuhan University, Wuhan, Hubei, China; ^2^ Department of Gastroenterology, Renmin Hospital of Wuhan University, Wuhan, Hubei, China

**Keywords:** microbiota, colorectal cancer, screening, bibliometric, citespace

## Abstract

**Aims:**

We conducted bibliometric and visualization analyses to evaluate the current research status, hotspots, and trends related to the human microbiota markers in colorectal cancer screening.

**Methods:**

The related studies were acquired from the Web of Science Core Collection (WoSCC) database on 5 January 2023. Analyses of the co-occurrence and cooperation relationships between the cited authors, institutions, countries/regions, cited journals, cited articles, and keywords in the studies were carried out using CiteSpace 5.8.R3 software and the Online Analysis platform of Literature Metrology. Additionally, relevant knowledge graphs were drawn to perform visualization analyses; a keywords cluster analysis and a burst analysis were also conducted.

**Results:**

After analyzing 700 relevant articles, this bibliometric analysis found that the annual publications showed an increasing trend from 1992 to 2022. Yu Jun from the Chinese University of Hong Kong had the highest cumulative number of publications, whereas Shanghai Jiao Tong University was the most productive institution. China and the USA have contributed the largest number of studies. The keywords frequency analysis demonstrated that “colorectal cancer,” “gut microbiota,” “*Fusobacterium nucleatum*,” “risk,” and “microbiota” were the most frequent keywords, and the keywords cluster analysis found that the current hotspots were as follows: (a) the precancerous lesions of colorectal cancer (CRC) that need to be screened, such as inflammatory bowel disease (IBD) and advanced adenoma, (b) the gut-derived microbiome for CRC screening, and (c) the early detection of CRC. The burst analysis further showed that the combination of microbiomics with metabolomics might be the future research trend in the field of CRC screening.

**Conclusion:**

The findings of the current bibliometric analysis firstly provide an insight into the current research status, hotspots, and future trends in the field of CRC screening based on the microbiome; the research in this field is becoming more in-depth and diversified. Some human microbiota markers, especially “*Fusobacterium nucleatum*,” are promising biomarkers in CRC screening, and a future hotspot might be the combined analysis of microbiomics and metabolomics for CRC risk screening.

## 1 Introduction

Colorectal cancer (CRC) is one of the most common cancers, and its morbidity and mortality rank third and second, respectively, among all types of malignant tumors. According to statistical data in 2020, the numbers of new CRC cases and deaths were up to 1,880,700 and 915,800, respectively, which posed a great burden to health systems across the world (1). In recent years, with the progress of the research on CRC, great advances have been made in its treatment; however, it remains difficult to cure CRC, especially for patients with advanced CRC. The prognosis of CRC is closely correlated with the clinical stage at diagnosis: The 5-year survival rate of stage I is up to 90%, whereas that of advanced CRC with distant metastasis is merely 14% (2). Former studies have shown that population screening is an effective way to reduce the incidence and mortality of CRC (3, 4). As the early stage of CRC commonly has no obvious clinical symptoms, a method to detect CRC early is particularly important. The commonly used clinical screening methods for CRC include colonoscopy, guaiac fecal occult blood testing (gFOBT), a fecal immunochemical test (FIT), CT colonography (CTC), and CRC risk assessment models such as the Asia-Pacific Colorectal Screening (APCS) score, yet each of these methods has its own deficiencies (5, 6). Therefore, an ideal choice for CRC screening is still lacking.

The human microbiome is a complex ecosystem with an extremely rich variety and quantity of microbes. They mainly colonize the skin, mouth, lung, intestinal tract, and vagina, especially the gut and mouth, two of the largest microbial habitats with more than 1,000 types of colonization bacteria (7, 8). These microbes play an important role in maintaining host homeostasis, whereas microbial dysbiosis is also closely related to diseases of various systems, including the gastrointestinal system, endocrine system, central nervous system, and immune system (9). In recent years, microbiological research has also gradually become one of the hotspots in the field of CRC research. Accumulating evidence indicates that the development of CRC is associated with the human microbiome. In terms of the screening of CRC, a recently published study discovered five types of gut microbiota markers (*Intestinimonas butyriciproducens*, *Holdemania filiformis*, *Firimicutues bacterium CAG 83*, *Bilophilia wadsworthia*, and *Alistipes putredinis*) differentially enriched between CRC and healthy controls, and these microbiota markers might function as CRC risk screening biomarkers (10). In addition to gut-derived microbiota markers, Zhang C (11) et al. found a higher proportion of oral *Fusobacterium*, *Streptococcus*, and *Herbaspirillum* and a lower proportion of *Haemophilus* and *Neisseria* in patients with CRC than in healthy controls. They further developed a screening model for CRC by using a random forest algorithm and oral microbiota markers, and they showed that the area under curve (AUC) of this model for CRC screening was up to 0.96. Another study also found that anti-Fn-IgA and -IgG in the serum of patients with CRC were higher than those in patients with benign colorectal disease and healthy individuals (*P* < 0.001) (12), suggesting that blood microbiota markers also have a potential ability to detect CRC. The value of these microbiota markers, especially *Fusobacterium*, in CRC screening has also been intensively investigated in many other studies (13–15). The results of those studies provide novel ideas for CRC screening; thus, analyzing the current research status, hotspots, and study trends in the field of CRC screening based on the microbiome brings great reference value for CRC screening in clinical practice and further basic research.

Due to the rapid growth of the literature on the topic of CRC screening based on the microbiome, it is difficult to conduct a comprehensive assessment of this research field by manually retrieving documents. Bibliometrics takes studies as the research object, and it evaluates the current study status and future research trends of a certain field by conducting qualitative and quantitative analyses; these processes are mainly based on the keywords, authors, institutions, countries, source journals, and other information of the selected studies (16). Different from traditional citation counting, bibliometrics focuses on the connection between studies; thus, it performs well in creating knowledge structures and predicting emerging trends (17, 18). Nowadays, as an emerging method, bibliometrics has been used in many disciplines, including medicine (19–22). However, to the best of our knowledge, there are still no available bibliometric analysis studies that evaluate the current research status, hotspots, and trends related to the human microbiota biomarkers in CRC screening worldwide. Thus, we conducted bibliometric and visualization analyses to clarify the research status and future trends of human microbiota biomarkers in the detection of CRC.

## 2 Materials and methods

### 2.1 Data source and retrieval strategy

Due to the characteristics of a bibliometric analysis, no ethical issues were involved in this study; therefore, approval from an ethics committee or institutional review board was not required. We completed the literature search according to the criteria of search strategies on 5 January 2023. The database of the Web of Science Core Collection (WoSCC) has a rigorous assessment process and can provide influential and credible information, so we chose WoSCC (https://clarivate.com/) as the source of data. The retrieval time range was not limited, the language was restricted to English, and the retrieved article type was limited to research studies and review papers. We applied the subject term search method as the retrieval strategy in the database. The document search was performed by using the following search formula: “[TS = (colorectal cancer) or TS = (colorectal neoplasm) or TS = (colorectal carcinoma) or TS = (colorectal tumor)] AND [TS= (microbiota) or TS = (microbiome) or TS = (bacteria)] AND [TS = (screening) or TS = (screen) or TS = (detect) or TS = (detection)]”. Two independent data collectors read the titles and abstracts of the studies retrieved from the database, and any disagreements were settled by discussion. When the discussion failed to solve the differences between the two data collectors, a third author was invited to participate in the discussion and consensus.

### 2.2 Data refinement and extraction

The document management software program called Note Express was used to manage the articles retrieved from the database. The articles’ information was imported into Note Express, and the software removed the redundant publications. After two authors carefully checked the remaining papers, the total number of articles finally included in the present study was 700. The metadata file exported from WoSCC was regarded as the bibliographic analysis information file, and it was saved in the format of “RefWorks.” Two researchers independently reviewed the included studies. The extracted data used for the follow-up analysis were as follows: (a) article title, (b) the full names of the authors, (c) publication year, (d) authors’ institutions and counties/regions, (e) keywords, (f) the total number of citations, (g) topics, (h) cited articles, and (i) the names of published journals and corresponding impact factors (IFs) according to the latest published Journal Citation Reports (JCRs).

### 2.3 Bibliometric data analysis and visualization

We saved the extracted data file in the format of “txt” and named it “download_*.txt.” Then, the file was imported into CiteSpace 5.8.R3 (Drexel University, Philadelphia, PA, USA), software for a knowledge map visual analysis, developed by Professor Chen Chaomei from Drexel University. This software helped us to extract relevant information from the included articles and visualize the key information in the form of nodes and links. CiteSpace has been applied in various fields (23). At the same time, we also imported the “txt” format data file into the Online Analysis Platform (http://bibliometric.com/) for a bibliometrics analysis. This platform is an intuitive, user-friendly website, where bibliometric data analyses of scientific citation data can be presented visually in graphics.

CiteSpace was used to visualize the co-occurrence network for authors, institutions, counties/regions, journals, articles, and keywords. Additionally, a cluster analysis and burst detection were also conducted on the keywords. In this study, the parameter settings applied in CiteSpace were as follows: (a) time span: from the inception of the database to 31 December 2022; (b) years per slice: 1 year; (c) method used to evaluate relationship strength: cosine; (d) node types: author, cited author, institution, county, reference, and keyword; (e) filter criteria: top 50 in each time interval; and (f) pruning network: pathfinder, pruning sliced networks, and pruning the merged network. We selected the default parameters of CiteSpace for the remaining settings. The Log-likelihood ratio (LLR) algorithm was used for the cluster analysis of the main keywords. Two evaluation indices, namely, Modularity Q and Weighted Mean Silhouette S, were calculated to evaluate the effect of the cluster analysis. The interval of the *Q* value was between 0 and 1, and this indicator demonstrated the goodness of the network structure. Normally, a *Q* value higher than 0.3 means that the structure of the clustering network is significant. The *S* value ranged from −1 to 1, and the *S* value was positively related to the rationality of the clustering network. An *S* value higher than 0.5 indicates that the clustering network is reasonable, whereas a value higher than 0.7 indicates that the clustering result is convincing. Burst detection was used to detect emergent keywords with a high-frequency change rate and a fast growth rate by elucidating the time distribution of the keywords, which could help us understand the research hotspots and trends of CRC detection based on the microbiome. Furthermore, we also used the Online Analysis Platform to investigate the number of common national articles; the keywords per year; the relationship between authors, institutions and countries; and the citation relationship between articles.

## 3 Results

### 3.1 Annual scientific publications and general characteristics

**Figure 1:**
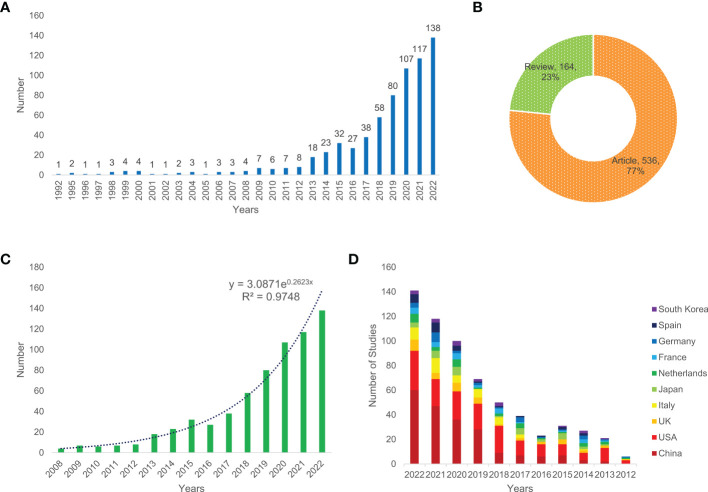
Annual number of publications **(A)**, the type of literature **(B)**, exponential growth graph of number of publications in the past 15 years **(C)**, and the number of publications per year in common countries in the past 11 years **(D)** of relevant literature.

After removing other types of articles, such as conference abstracts, editorial material, book chapters and letters, a total of 700 papers, comprising 536 (77%) articles and 164 (23%) reviews (**Figure 1B**), related to CRC screening based on the microbiome were included in this bibliometric analysis. **Figure 1A** shows that the number of annual publications increased from 1992 to 2022 and that the highest number of publications was in 2022; up to 138 studies were published in this year, implying that the research on CRC screening based on the microbiome has attracted increasingly more attention. The publishing of articles increased exponentially according to Price’s law, and the mathematical formula of the exponential curve equation is *y* = 3.0871e^0.2623x^. **Figure 1C** shows that the simulation curve is in good agreement with the yearly publication growth trend, and the coefficient of determination increased to 0.9748, indicating that the number of new articles published annually will continue to increase in the coming years. Additionally, the average annual rate of increase was 26.23% for the past 15 years. In terms of the trend of the number of published articles in common countries/regions, China and the USA were the two countries with the largest number of published papers (**Figure 1D**).

### 3.2 Analysis of cited authors

**Figure 2:**
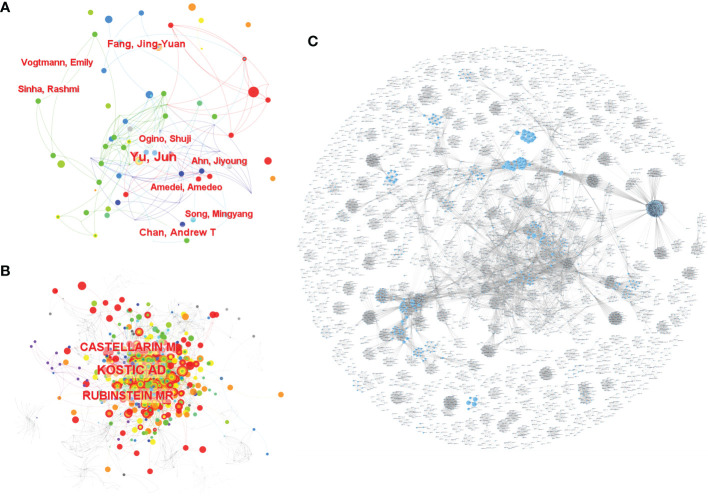
The co-occurrence of authors **(A)** and cited authors **(B)** and the cooperation relationship between authors **(C)** of relevant literature. [**(C)** Each small blue dot represents an author, the link represents collaboration, and the larger the blue dot, the more collaboration].

A total of 4,752 authors conducted the relevant studies obtained from the WoSCC in the field of CRC screening based on the microbiome (**Supplementary Table S1**). A cooperation network map of author and cited authors was constructed using CiteSpace. A co-occurrence network knowledge map of author was also constructed using CiteSpace. A total of 616 nodes representing 616 authors were illustrated in the co-occurrence network map (**Figure 2A**). The size of the nodes was positively related to the number of papers published by the author, of whom the author Yu Jun from the Chinese University of Hong Kong had the highest cumulative number of publications, indicating that they have made notable contributions to the field of CRC screening based on the microbiome. The high citation index (h-index) is an easy-to-use index for the evaluation of the influence of researchers’ work, and it is determined by the number of highly influential published studies. Normally, a high h-index means that a researcher has a high academic impact. In this bibliometric analysis, the author Ogino Shuji from Harvard University had the highest h-index with 98 (**Table 1**); thus, they are the most influential among all researchers in this field. We also analyzed the cited authors by using CiteSpace, and there were 996 cited authors in total. Kostic A.D., Castellarin M., and Rubinstein M.R. ranked as the top three authors for the frequency of citations (**Figure 2B** and **Supplementary Table S2**). Furthermore, the cooperation network diagram was applied to explore the cooperation between the author clusters, and it could be seen in the diagram that more than half of the author clusters had less cooperation with others (**Figure 2C**).

**Table 1:** Top 10 authors of relevant literature based on CiteSpace.

Rank	Authors	Institutions	Frequency	H-index	Median citation percentile	Degree
1	Yu Jun	Chinese University of Hong Kong	9	89	83^rd^	7
2	Chan Andrew T	Fred Hutchinson Canc Res Ctr	5	64	82^nd^	8
3	Fang Jing-Yuan	Shanghai Jiao Tong University	5	59	67^th^	8
4	Ahn Jiyoung	New York University	4	45	80^th^	10
5	Song Mingyang	Harvard University	4	42	76^th^	8
6	Vogtmann Emily	National Cancer Institute	4	25	77^th^	7
7	Ogino Shuji	Harvard University	4	98	85^th^	7
8	Sinha Rashmi	National Institute of Technology	4	37	86^th^	6
9	Amedei Amedeo	University of Florence	4	48	70^th^	5
10	Nosho Katsuhiko	Harvard University	3	57	83^rd^	11

### 3.3 Analysis of institutions and countries/regions

**Figure 3:**
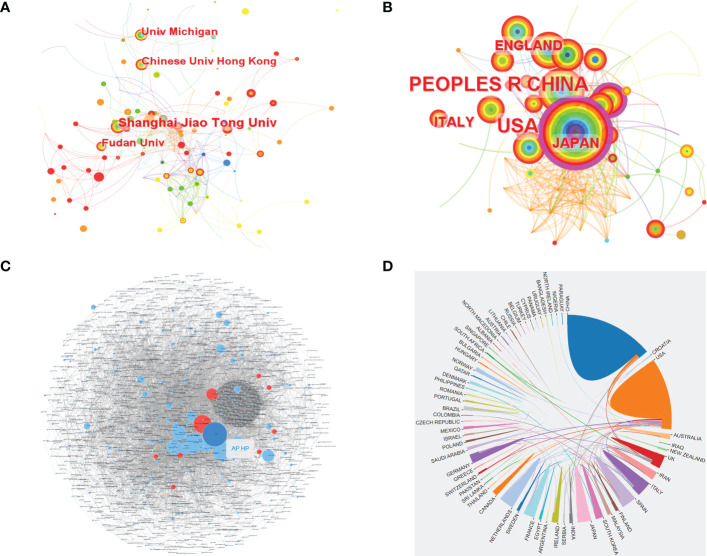
The co-occurrence of institutions **(A)** and countries **(B)**, and the cooperation relationship between institutions **(C)** and countries **(D)** of relevant literature. [**(C)** Each small blue dot represents an institution, the link represents collaboration, and the larger the blue dot, the more collaboration. The dark blue dot is the institution that cooperates the most, and the red dots are the institutions that cooperate with that institution].

Based on WoSCC, a total of 1,448 institutions and 71 countries/regions contributed articles related to CRC screening based on the microbiome. Among them, Shanghai Jiao Tong University ranked first with 26 publications, and China had the largest number of publications, reaching 214. The USA (contributed 186 articles) was one of the two countries (China and the USA) that produced more than 100 articles (**Supplementary Table S1**). By using CiteSpace, we also analyzed the cooperation networks of the institutions and countries/regions, and network relationship diagrams of the co-occurrence of institutions and countries/regions were also visualized. Based on CiteSpace, it was found that there were 398 institutions published relevant articles on the topic, and the top 4 most productive institutions were Shanghai Jiao Tong University, Fudan University, the Chinese University of Hong Kong and the University of Michigan, with 23, 15, 14, and 13 publications, respectively (**Figure 3A** and **Supplementary Table S2**). **Figure 3C** shows the cooperation relationships between the institutions, and these relationships were determined using the Online Analysis Platform. Every small dot represents a corresponding article, whereas mutual citations are denoted by the lines between the dots. In this figure, we could see that there was a close connection between the institutions. In terms of the analysis of the countries/regions, there were 71 countries/regions included according to CiteSpace. Among all these countries/regions, China was the most productive country in terms of publishing articles on CRC screening based on the microbiome. The number of publications from China was up to 207, followed by the USA with 180 articles, Italy with 45 articles, Japan with 39 articles and England with 35 articles (**Figure 3B** and **Supplementary Table S2**). However, the diagram of the cooperation between countries/regions demonstrated that China had less cooperation with other countries/regions, whereas the USA cooperated more closely with other countries/regions than China (**Figure 3D**).

### 3.4 Analysis of cited journals and cited articles

**Figure 4:**
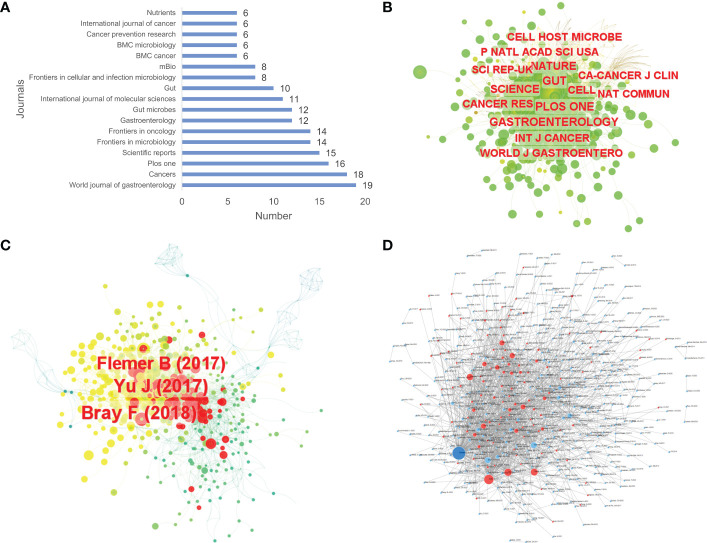
The number of articles published by journals **(A)**, the co-occurrence of cited journals **(B)** and cited articles **(C)**, and the article citation relationship network **(D)** of relevant literature. [**(D)** Each small blue dot represents an article, the links represent citations, and the larger the blue dot, the more citations. The dark blue dot is the most cited article, and the red dots are the articles that cite that article].

By using WoSCC, it was found that up to 368 journals published relevant papers on the topic. *World Journal of Gastroenterology* contributed the largest number of publications with 19 articles (**Figure 4A**). By using CiteSpace, analyses of the cooperation networks of the cited journals and cited articles were performed, and co-occurrence visualization maps were also presented. There were 698 cited journals in total, of which the *Gut* journal ranked first in terms of the frequency of citations, reaching 441 citations, and up to 14 journals were cited over 200 times (**Figure 4B** and **Supplementary Table S2**). Regarding cited articles, 1,011 articles were cited. The study conducted by Bray F and others (24) was the most cited among all cited articles with 68 citations, followed by the articles published by Yu J et al. (25) with 61 citations and Flemer B et al. (26) with 57 citations (**Figure 4C**). By using the Online Analysis Platform, an article citation network diagram was also drawn. Every small dot represents a corresponding article, whereas mutual citations are represented by the lines between the dots. In this diagram, we could see that most of the 700 articles were cited from each other, and a study published in *Genome Research* was cited the most frequently (27) (**Figure 4D**).

### 3.5 Keywords analysis

#### 3.5.1 Word frequency analysis

**Figure 5:**
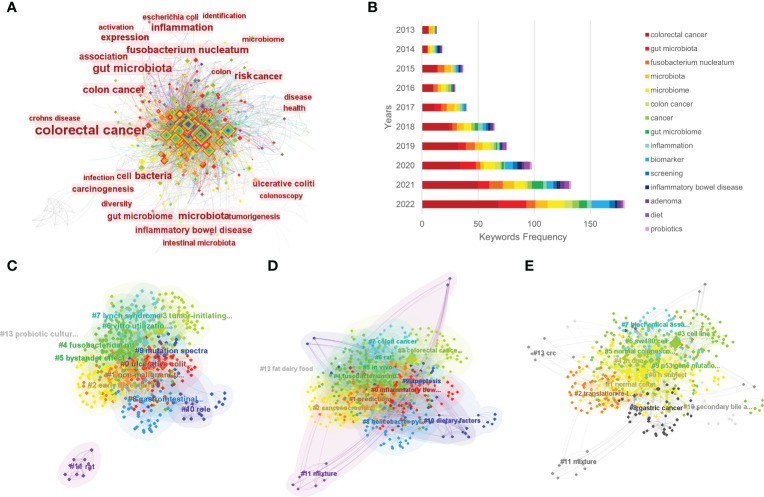
The co-occurrence of keywords **(A)**, annual number of common keywords **(B)**, and the clustering maps based on title words **(C)**, keywords **(D)**, and abstracts **(E)** of relevant literature.

Keywords are a brief summary of the research content, reflecting the key information of the article. In a bibliometric analysis, keywords can be used to explore the hotspots and trends in a certain field (28). By using CiteSpace, a co-occurrence visualization map of the keywords was developed, and 628 nodes were presented. Every node represents a corresponding keyword, and the lines between two nodes represent the relationships between the keywords. The cool colors of the connection lines indicate the earlier appearance of the keywords, whereas the warm colors indicate their later appearance. Additionally, the thickness of the connection lines represents the frequency of co-occurrence (**Figure 5A**). Furthermore, we visualized the keywords’ frequencies year by year, as shown in **Figure 5B**, and the frequency has maintained a growth trend over the past 10 years. In **Table 2**, we can see the occurrence frequency of the top 15 keywords, of which, “colorectal cancer” ranked first with 374 times, followed by “gut microbiota,” “*Fusobacterium nucleatum*,” “risk,” and “microbiota.” We also analyzed the centrality of keywords; centrality is a measurement index of the role of the nodes in a whole network, and a higher centrality routinely implies that the keyword has a larger influence in the field. The top three keywords in terms of centrality were “colorectal cancer,” “colon cancer,” and “bacteria” (**Table 2**).

**Table 2:** Top 15 keywords of relevant literature.

Rank	Keywords	Frequency	Keywords	Centrality	Keywords	Degree	Keywords	Burst
1	colorectal cancer	374	colorectal cancer	0.38	colorectal cancer	148	colon cancer	5.03
2	gut microbiota	154	colon cancer	0.19	colon cancer	103	inflammatory bowel disease	4.65
3	fusobacterium nucleatum	88	bacteria	0.12	bacteria	89	Escherichia coli	4.15
4	risk	79	cancer	0.11	cancer	79	Metabolite	4.12
5	microbiota	76	inflammatory bowel disease	0.1	inflammatory bowel disease	78	ulcerative colitis	4.03
6	colon cancer	75	expression	0.09	expression	72	Association	3.93
7	inflammation	68	carcinogenesis	0.09	association	71	Microbiota	3.81
8	bacteria	65	Escherichia coli	0.08	carcinogenesis	70	fusobacterium nucleatum	3.7
9	cancer	53	bile acid	0.08	Escherichia coli	62	Crohn’s disease	3.65
10	expression	52	gut microbiota	0.08	cell	56	tumorigenesis	3.43
11	inflammatory bowel disease	47	association	0.07	bile acid	56	epithelial cell	3.28
12	cell	47	inflammation	0.07	ulcerative colitis	55	human gut microbiome	3.23
13	association	43	DNA	0.06	gut microbiota	54	African American	3.13
14	gut microbiome	43	gene expression	0.06	inflammation	53	prevalence	3.1
15	carcinogenesis	38	gut	0.06	tumorigenesis	53	island methylator phenotype	3.04

#### 3.5.2 Cluster analysis

**Figure 6:**
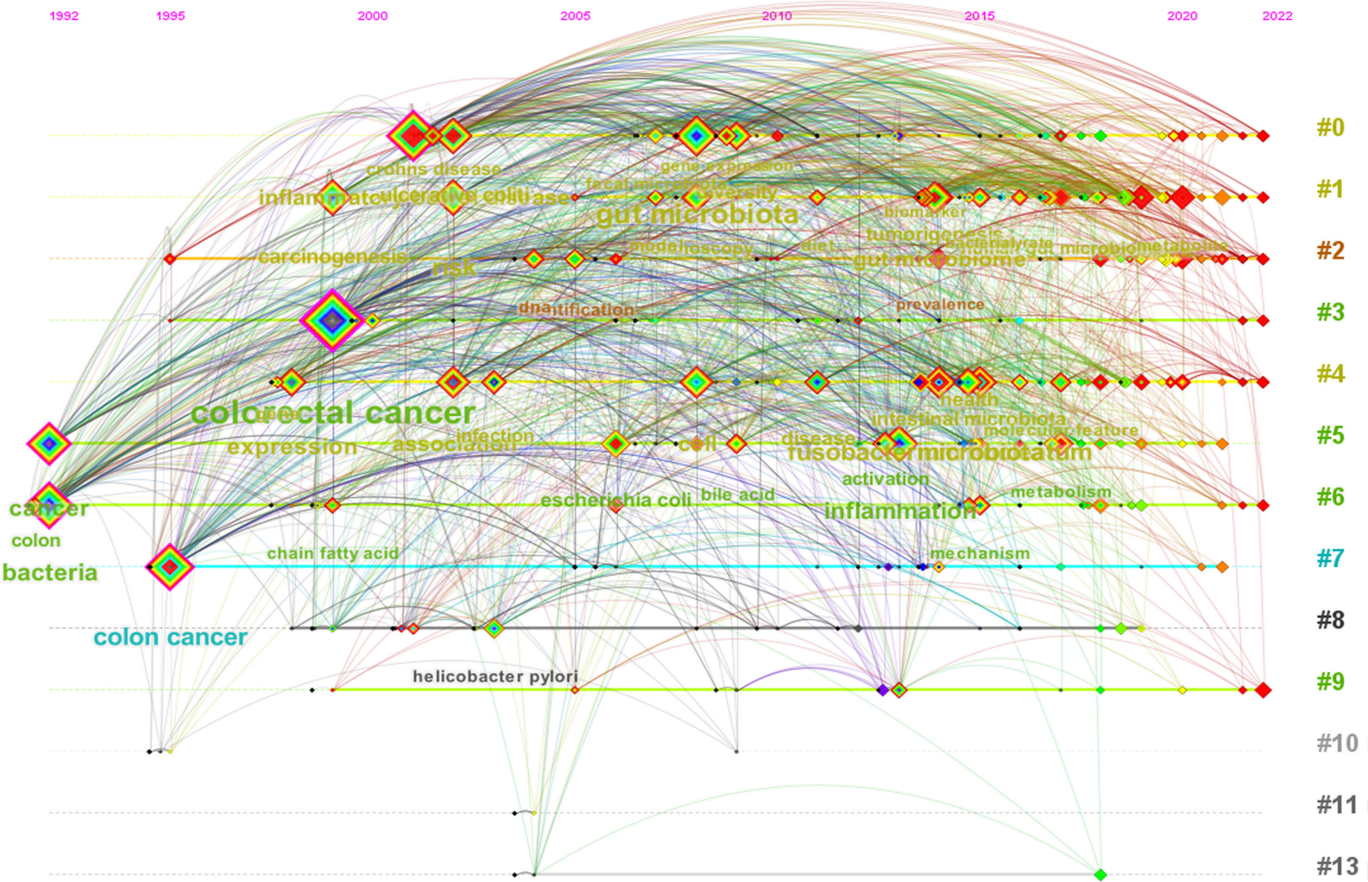
The clustering timeline view of keywords of relevant literature.

Using CiteSpace software, we performed a cluster analysis based on the title words, keywords and abstracts. The cluster diagrams are shown in **Figures 5C–E**. Each color block area in the cluster diagrams represents a cluster, and there are 13 clusters formed in the figures. The clusters were encoded from #0 to #13 (**Table 3**); a smaller number meant that more keywords were included in the clusters. Multiple overlapping areas were also found among the different color blocks in the cluster diagrams, indicating that those clusters had close relationships. A *Q* value of 0.475 (> 0.3) and an *S* value of 0.7689 (> 0.7) further indicated that the clustering result was informative and reasonable. Additionally, a clustering timeline diagram was also obtained (**Figure 6**), and this diagram visualizes the time period of the clusters and the relationships among the different clusters. From the cluster analysis in this study, we determined that the current hotspots in this field were mainly focused on the following three aspects: (a) the precancerous lesions of CRC that need to be screened, such as inflammatory bowel disease (IBD) and advanced adenoma; (b) the gut-derived microbiome for CRC screening; and (c) the early detection of CRC.

**Table 3:** Keywords co-occurrence network clustering table.

Category	Cluster ID	Size	Mean (year)	Top terms (top 5)
A. Based on title words	0#	77	2013	ulcerative colitis (442.81, 1.0E-4); inflammatory bowel disease (348.08, 1.0E-4); inflammatory bowel diseases (188.81, 1.0E-4); inflammatory status (171.21, 1.0E-4); old age (171.21, 1.0E-4)
	1#	70	2016	non-malignant tissue (173.42, 1.0E-4); normal participant (169.64, 1.0E-4); other intestinal disorder (169.64, 1.0E-4); clostridium butyricum (160.25, 1.0E-4); colorectal cancer gut microbiome (153.07, 1.0E-4)
	2#	65	2015	early detection (206.29, 1.0E-4); early onset colorectal cancer (178.12, 1.0E-4); non-invasive colorectal cancer (164.38, 1.0E-4); bacterial two-hybrid system (157.51, 1.0E-4); premature stop codon-detection method (157.51, 1.0E-4)
	3#	63	2008	tumor-initiating potency (134.72, 1.0E-4); phenolic-rich dietary fiber matrix (132.27, 1.0E-4); human renal cell carcinoma cell line (124.93, 1.0E-4); colonic carcinogenesis model (122.57, 1.0E-4); heterocyclic amine aminophenylnorharman (122.57, 1.0E-4)
	4#	62	2013	fusobacterium nucleatum (268.83, 1.0E-4); population-based cohort study (230.55, 1.0E-4); anaerobic bacteria (230.55, 1.0E-4); molecular feature (159.77, 1.0E-4); gastric cancer (139.99, 1.0E-4)
	5#	54	2012	bystander effect (152.51, 1.0E-4); enterococcus faecalis (152.51, 1.0E-4); metabolite perspective (148.67, 1.0E-4); high-fat diet-induced colitis-associated cancer (144.84, 1.0E-4); evodiamine inhibit (144.84, 1.0E-4)
	6#	53	2009	vitro utilization (182.14, 1.0E-4); intestinal bacteria (172.81, 1.0E-4); human colonic bacteria (170.92, 1.0E-4); high-amylose maize (170.92, 1.0E-4); tissue-associated microbiota (166.09, 1.0E-4)
	7#	52	2008	lynch syndrome (186.92, 1.0E-4); natural-history surveillance management (186.92, 1.0E-4); translesion DNA synthesis (179.41, 1.0E-4); deoxycytidine adduct (179.41, 1.0E-4); alpha-dehydroxylating clostridia desulfovibrio vulgaris methanobrevibacter (171.91, 1.0E-4)
	8#	51	2005	gastrointestinal tract (125.33, 1.0E-4); family history (115.02, 1.0E-4); clinical management (105.29, 1.0E-4); early stage (94.72, 1.0E-4); bacterial antigen (94.72, 1.0E-4)
	9#	25	2010	mutation spectra (90.85, 1.0E-4); new approaches (90.85, 1.0E-4); understanding p53 gene tumor (90.85, 1.0E-4); compound k (79.44, 1.0E-4); multiple pathway (79.44, 1.0E-4)
	10#	13	1996	role (19.19, 1.0E-4); bile-acid (19.19, 1.0E-4); colorectal carcinogenesis (8.24, 0.005); colorectal cancer (0.61, 0.5); fusobacterium nucleatum (0.14, 1.0)
	11#	11	2004	rat (18.01, 1.0E-4); mixture (18.01, 1.0E-4); cecal microbial metabolism (18.01, 1.0E-4); change (18.01, 1.0E-4); drinking water disinfection by-product (18.01, 1.0E-4)
	13#	9	2005	probiotic culture (33.72, 1.0E-4); fermented milk (33.72, 1.0E-4); mechanistic approach (16.73, 1.0E-4); nutraceutical (16.73, 1.0E-4); colon cancer (10.6, 0.005)
B. Based on keywords	0#	77	2013	inflammatory bowel disease (27.45, 1.0E-4); Crohn’s disease (23.01, 1.0E-4); ulcerative colitis (23.01, 1.0E-4); inflammatory bowel diseases (7.88, 0.005); tumor growth (7.88, 0.005)
	1#	70	2016	prediction (14.55, 0.001); adenoma (13.34, 0.001); machine learning (10.91, 0.001); 16s RNA (10.53, 0.005); gut microbiome (10.36, 0.005)
	2#	65	2015	cancer screening (15.68, 1.0E-4); liquid biopsy (15.68, 1.0E-4); microRNA (11.34, 0.001); cardiovascular disease (10.45, 0.005); circulating tumor cells (10.45, 0.005)
	3#	63	2008	colorectal cancer (27.26, 1.0E-4); f (7.33, 0.01); nanoparticles (7.33, 0.01); modulation (7.33, 0.01); colonoscopy (6.92, 0.01)
	4#	62	2013	fusobacterium nucleatum (34.03, 1.0E-4); expression (13.46, 0.001); anaerobic bacteria (10.86, 0.001); inflammatory bowel disease (10.36, 0.005); prognosis (7.24, 0.01)
	5#	54	2012	*in vivo* (16.97, 1.0E-4); Escherichia coli (16.41, 1.0E-4); DNA damage (12.22, 0.001); activation (11.6, 0.001); genomic instability (8.47, 0.005)
	6#	53	2009	rat (6.66, 0.01); gut bacteria (6.66, 0.01); transgenic mice (5.15, 0.05); slc26a3 (5.15, 0.05); subtractive genomics approach (5.15, 0.05)
	7#	52	2008	colon cancer (26.53, 1.0E-4); breast cancer (11.21, 0.001); clinical trial (8.1, 0.005); body mass index (8.1, 0.005); chemoprevention (8.1, 0.005)
	8#	51	2005	helicobacter pylori (26.41, 1.0E-4); gastric cancer (19.92, 1.0E-4); enteric bacteria (14.66, 0.001); intestinal metaplasia (10.89, 0.001); epidemiology (10.89, 0.001)
	9#	25	2010	apoptosis (10.07, 0.005); gut microbiota (8.48, 0.005); high nitrite diet (8.33, 0.005); p27mt (8.33, 0.005); GC-MS (8.33, 0.005)
	10#	13	1996	dietary factors (12.96, 0.001); bile acids and salts (12.96, 0.001); colonic carcinogenesis (12.96, 0.001); cell proliferation (10.19, 0.005); cytotoxicity (7.97, 0.005)
	11#	11	2004	mixture (12.96, 0.001); bacterial metabolism (12.96, 0.001); water (12.96, 0.001); disinfection (10.19, 0.005); colon (7.24, 0.01)
	13#	9	2005	fat dairy food (10.48, 0.005); antimutagenic property (10.48, 0.005); casei strain Shirota (10.48, 0.005); lactobacillus acidophilus (10.48, 0.005); nutraceutical (10.48, 0.005)
C. Based on abstracts	0#	77	2013	h subject (723.74, 1.0E-4); Desulfovibrio sp (672.3, 1.0E-4); i-deficient mice (522.7, 1.0E-4); Crohn’s disease (447.02, 1.0E-4); prausnitzii population (433.93, 1.0E-4)
	1#	70	2016	normal colon (748.2, 1.0E-4); colorectal neoplasia (589, 1.0E-4); dietary polyphenol (516.82, 1.0E-4); advanced adenoma (501.93, 1.0E-4); marker fn (493.31, 1.0E-4)
	2#	65	2015	translation re-initiation event (555.44, 1.0E-4); human gene (555.44, 1.0E-4); extracellular vesicle (380.14, 1.0E-4); lethal prostate cancer (343.91, 1.0E-4); baseline serum level (343.91, 1.0E-4)
	3#	63	2008	cell line (1344.4, 1.0E-4); tumor-bearing mice (767.96, 1.0E-4); kg body (679.71, 1.0E-4); corresponding tumor tissue (679.71, 1.0E-4); phenolic metabolite (660.79, 1.0E-4)
	4#	62	2013	Fn DNA (983.65, 1.0E-4); HNSCC tissue (952.31, 1.0E-4); saliva sample (807.06, 1.0E-4); fusobacterium species (747.77, 1.0E-4); CRC diagnosis (713.57, 1.0E-4)
	5#	54	2012	normal colonoscopy (568.57, 1.0E-4); intestinal microbiome (559.26, 1.0E-4); endoscopic finding (553.3, 1.0E-4); obese adult (455.98, 1.0E-4); high-fat diet (443.6, 1.0E-4)
	6#	53	2009	sw480 cell (833.23, 1.0E-4); amylopectin maize starch (593.32, 1.0E-4); Bifidobacterium spp (593.32, 1.0E-4); peptide m2163 (499.5, 1.0E-4); FMT treatment (457.84, 1.0E-4)
	7#	52	2008	biochemical assay (444.15, 1.0E-4); metachronous CRC (321.72, 1.0E-4); human cell (308.83, 1.0E-4); degrees c (295.96, 1.0E-4); IBD-associated CRC (283.07, 1.0E-4)
	8#	51	2005	gastric cancer (560.39, 1.0E-4); MUC gene expression (418.01, 1.0E-4); mucosal surface (313.36, 1.0E-4); colorectal cancer (305.79, 1.0E-4); mucus gel (208.79, 1.0E-4)
	9#	25	2010	p53 gene mutation (233.83, 1.0E-4); human cancer (155.79, 1.0E-4); human tumor (155.79, 1.0E-4); panax ginseng (146.04, 1.0E-4); cell cycle (126.56, 1.0E-4)
	10#	13	1996	secondary bile acid (50, 1.0E-4); fruit intake (16.63, 1.0E-4); genetic susceptibility (16.63, 1.0E-4); metabolism (16.63, 1.0E-4); dehydroxylation (16.63, 1.0E-4)
	11#	11	2004	mixture (81.06, 1.0E-4); change (48.51, 1.0E-4); treatment (41.79, 1.0E-4); intestinal microbial metabolism (32.3, 1.0E-4); DBP (32.3, 1.0E-4)
	13#	9	2005	CRC (96.83, 1.0E-4); probiotics (82.96, 1.0E-4); milk (82.96, 1.0E-4); evidence (77.22, 1.0E-4); probiotic culture (55.27, 1.0E-4)

#### 3.5.3 Burst analysis

The emerging words in the literature about the microbiota in CRC screening were analyzed using CiteSpace. The years’ distributions of each emerging word are presented in the form of numbers and red bars. The begin time and the end time are also displayed, and the frequency of the emerging words during their emergence time is presented with their strength; a higher strength is related to a higher frequency (**Table 4**). In the early period of 1992-2022, the research was mainly focused on pathophysiological states, such as IBD and gene mutations, which might induce CRC by altering the human microbiome. In the midterm period, the research was mainly focused on specific human bacteria, such as *Escherichia coli* and *Fusobacterium nucleatum*, which may function as potential biomarkers for CRC. In the later period, the research was mainly focused on the gut microbiome or metabolomics as the biomarkers of CRC risk screening. It was notable that research on the combination of the microbiome and metabolomics might be the future research trend in the field of CRC screening.

**Table 4:** Emergent analysis of keywords in relevant literature.

Keywords	Strength	Begin	End	1992–2022
mutation	3.03	1995	2006	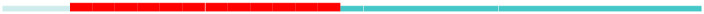
Crohn’s disease	3.65	2002	2015	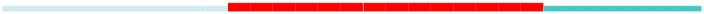
inflammatory bowel disease	4.65	2009	2016	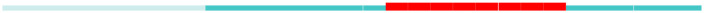
gene expression	2.82	2009	2014	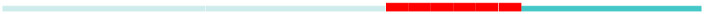
epithelial cell	3.28	2010	2017	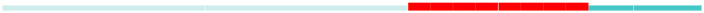
ulcerative colitis	4.03	2011	2015	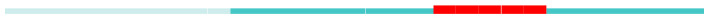
colon cancer	5.03	2013	2015	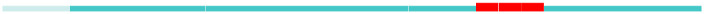
tumorigenesis	3.43	2014	2017	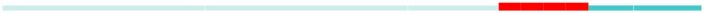
island methylator phenotype	3.04	2014	2018	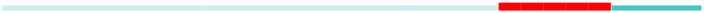
mice	2.61	2014	2015	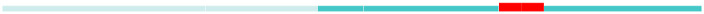
Escherichia coli	4.15	2015	2017	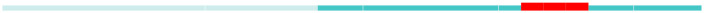
neoplasia	2.83	2015	2018	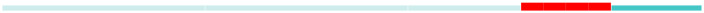
African American	3.13	2016	2018	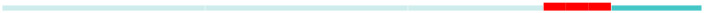
DNA damage	2.44	2016	2018	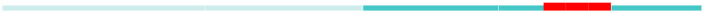
association	3.93	2017	2018	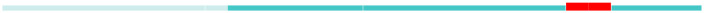
fusobacterium nucleatum	3.7	2017	2019	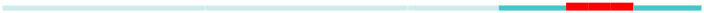
human gut microbiome	3.23	2017	2020	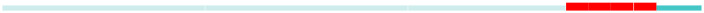
prevalence	3.1	2017	2019	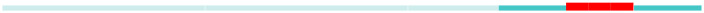
metabolism	2.58	2017	2019	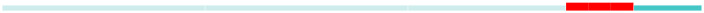
microbiota	3.81	2018	2019	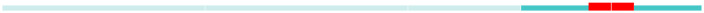
dietary fiber	2.98	2019	2022	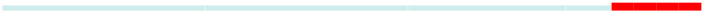
metabolite	4.12	2020	2022	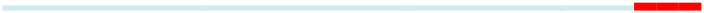
microbiome	2.99	2020	2022	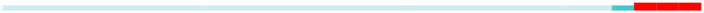
faecalibacterium prausnitzii	2.83	2020	2022	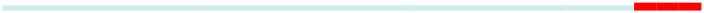
risk factor	2.43	2020	2022	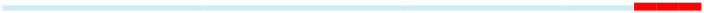

γ[0,1] = 0.7; minimum duration = 1.

## 4 Discussion

In the current study, we used the bibliometrics method and visualization tool to carry out an analysis of 700 selected studies related to CRC screening based on the microbiome, and we explored the current research status, hotspots and future trends after analyzing the roles played by the cited authors, institutions, counties/regions, cited journals, cited articles and keywords. To the best of our knowledge, this was the first bibliometric analysis that focused on the global research of microbiota biomarkers in CRC screening.

Since microbiological research has become one of the hotspots in the field of CRC research, accumulating evidence regarding the prevention, diagnosis, treatment and pathogenesis of CRC, among many other aspects, indicates that CRC is closely associated with the human microbiome. Furthermore, this has also attracted some researchers to conduct a comprehensive assessment in this research field. A recently published study examined up to 5,696 publications, and a bibliometric analysis found that “microbiome sequencing and tumor”; “microbiome compositions, interactions, and treatment”; “microbiome molecular features and mechanisms”; and “microbiome and metabolism” were the most intensively researched topics in the field of the gut microbiome in CRC research (29). Wu W (30) et al. also investigated the research trends in the relationship between the gut microbiome and CRC research; they found that “Gut Microbiota,” “Colorectal Cancer,” “Inflammation,” “Probiotic,” and so forth were the most frequent, and they revealed the current hotspots and trends in this field. However, the majority of the studies examined in both of the aforementioned studies were not related to CRC screening. Thus, through the two abovementioned studies, it might be difficult to accurately evaluate the current research status, hotspots, and trends in the field of CRC screening based on the human microbiome. Different to the two abovementioned studies, our bibliometric analysis only included publications that focused on microbiota biomarkers in CRC screening. In addition to gut microbiota, we also included studies that explored the potential value of the microbiota of the oral cavity, blood and other parts of the human body in CRC screening (12, 31, 32). Our study firstly provided an insight into the current hotspots and trends in the field of CRC screening based on the human microbiome. Due to the rapid growth of studies on the topic of CRC screening based on the human microbiome, we think that our bibliometric analysis is meaningful and brings great reference value for CRC screening in clinical practice and further basic research.

In this bibliometric analysis, we found that the number of yearly publications increased over the past 30 years. This trend suggests that researchers around the world became more interested in this field. Among the counties that contributed relevant studies, China and the USA ranked as the top 2 in terms of the number of publications, followed by Italy, Japan, UK, Germany, and France, showing that these countries have made advances in this field, and this might be the result of government-funded investment in medical research, as most of the countries are developed countries. China was the only developing country with the largest number of publications. This phenomenon might be explained by the rapid growth of financial investment by the Chinese government; this investment has even surpassed that of other countries, except for that of the USA. Moreover, China has the largest population in the world, which also provides a sufficient source of patients for clinical research, and these factors might have led to the great progress in the medical research of China (33, 34). Regarding the USA, the USA has the largest world economy in terms of the nominal gross domestic product (GDP), and it has advanced medical research systems with top medical institutions and researchers. These factors ensure that the USA has the ability to provide sufficient financial resources and research conditions for the study of microbiota in CRC screening (35), and they might clarify the reason why the USA has also contributed a relatively large number of publications. Notably, although China was the most productive in the field of CRC screening based on the microbiome, it did not cooperate much with other countries/regions in this field; therefore, China needs to strengthen its cooperation with other countries/regions. Consistent with the number of articles published by the various countries, the institution that ranked first in terms of the number of publications was Shanghai Jiao Tong University in China. Most institutions with a high number of publications were the top universities in their countries, and close inter-agency cooperation was generally observed.

In terms of the author analysis, a Chinese author named Yu Jun had the largest number of publications. Regarding academic influence, evaluated using the h-index, the USA author Ogino Shuji obtained the highest h-index of 98, whereas Yu Jun ranked second with an h-index of 89. Thus, we thought that Ogino Shuji was the most influential scholar, whereas Yu Jun was the most productive scholar with a high influence. Our bibliometric analysis found that there was less cooperation between the author clusters, so various author clusters should strengthen their collaboration in order to generate more breakthroughs in this field. Among all journals that published relevant articles, *World Journal of Gastroenterology* contributed the largest number of publications, followed by *Cancers*; the former is a professional journal in the field of research on digestive system diseases, whereas the latter is an oncology research journal. Regarding cited journals, *Gut* with the latest IFs of 31.79 ranked first in terms of the number of citations, and this indicates the high academic influence of *Gut* in this research field. Notably, in the analysis of the cited articles in this study, it was found that the article published in 2012 and entitled “*Fusobacterium nucleatum* infection is prevalent in human colorectal carcinoma” (27) was cited the most; this study used the quantitative real-time polymerase chain reaction (q-PCR) to amplify the DNA of *Fusobacterium nucleatum*, and it found an overabundance of the *Fusobacterium nucleatum* DNA sequence in tumor tissue compared with normal control tissue. The enrichment of *Fusobacterium nucleatum* was also positively correlated with lymph node metastasis, and the results of this study implied that fecal *Fusobacterium nucleatum* might be a potential biomarker in CRC screening. Furthermore, this was further verified by multiple studies published later. A meta-analysis published in 2019 assessed the diagnostic accuracy of fecal *Fusobacterium nucleatum* for CRC; it found that the sensitivity and specificity of fecal *Fusobacterium nucleatum* in CRC screening were 71 and 76%, respectively, and the area under the receiver‐operating characteristic (AUC) curve was 0.80. In addition to fecal *Fusobacterium nucleatum*, some studies have found that oral-derived *Fusobacterium nucleatum* is also a promising biomarker for CRC screening (36, 37).

A visualization of the keywords in the articles related to CRC screening based on the microbiome showed that the hot keywords were “colorectal cancer,” “gut microbiota,” “*fusobacterium nucleatum*,” “risk,” and “microbiota,” indicating that human gut-derived microbiota was the most commonly studied in this field. Among the gut microbiota, our analysis of keywords found that an anaerobic bacterium called *Fusobacterium nucleatum*, as mentioned above, played the role of “star intestinal bacteria” in CRC microbial markers. Gut-derived samples, such as fecal samples, are easy to obtain and noninvasive. Studies on the fecal microbiome in CRC risk assessment have been performed for many years, and relatively more articles are being published (13). Since the prognosis of CRC is closely correlated with the clinical stage at diagnosis, it is particularly important to detect CRC at an early stage. An increasing number of researchers have evaluated the potential role of the fecal microbiome in the early stages of colorectal cancer screening. Our cluster analysis also confirmed that the early detection of CRC has become one of the research hotspots. Among these published articles, the vast majority of publications investigated the performance of the fecal-derived microbiome in detecting advanced adenomas or polyps, and the AUC ranged from 0.28 to 0.87 (38–43). Some studies also evaluated the ability of the fecal-derived microbiome to detect stage I/II CRC, and the AUC ranged from 0.59 to 0.96 (44–47). To improve the screening effectiveness, some researchers combined the fecal microbiome with fecal occult blood (48–50), tumor markers [mainly the carcinoembryonic antigen (CEA)] (44, 50) and basic demographic information [such as age, sex, and body mass index (BMI)] (51, 52), and these combinations resulted in the improvement of the CRC screening ability.

In our burst analysis, we found that research on the combined analysis of microbiomics and metabolomics might be the future research trend in the field of CRC risk screening. Accumulating studies revealed that the gut microbiome and its metabolites were associated with colorectal tumorigenesis. *Escherichia coli* has been found to induce DNA methylation by producing trimethylamine, which might lead to the occurrence of CRC (53), whereas *Bilophila wadsworthia* and *Pyramidobacter spp* might induce CRC through the production of genotoxic hydrogen sulfide—a substance that has been found to enhance carcinogenesis in the gut (54–56). In contrast, a high-fiber diet has been shown to increase gut short-chain fatty acids (SCFAs), which protect the gut by reducing intestinal inflammation and lowering the risk of CRC. In this process, certain gut probiotics, such as *Roseburia*, *Bifdobacterium*, and *Lactobacillus*, play key roles in metabolizing dietary fiber into SCFAs (57, 58). A recently published study by Po-Li Wei et al. (40) combined gut microbiomics and metabolomics to construct a CRC screening model, and they demonstrated that the AUC of this model was 0.9155 in CRC screening; this AUC value was higher than that obtained with gut microbiomics only and metabolomics only. In another study, the researchers developed a microbe–metabolite diagnostic panel, and they found that the AUC was up to 0.994 for CRC and 0.912 for gut adenoma (47). Overall, combining metabolomics with microbiomics significantly improves the accuracy of CRC detection, so we thought that one of the potential breakthroughs in this field might be an individualized and accurate screening of CRC by using multiomics technology that includes but is not limited to human microbiomics and metabolomics in the future. However, there were relatively few related reports regarding CRC screening using a combined analysis of microbiomics and metabolomics and many unexplored problems need to be resolved in this field.

Our bibliometric analysis showed that the use of human microbiota markers might be a novel approach in CRC screening. Many studies also explored the potential mechanism of action of the human microbiota in CRC, explaining the reasons why the human microbiota could function as promising biomarkers for CRC screening. The mechanism of the human microbiota in CRC is complex, but it is mainly related to inflammation, immunization, genotoxins, oxidative stress, and bacterial metabolites (59, 60). Regarding inflammation and immunization, *Fusobacterium nucleatum*, *Porphyromonas gingivalis*, *Prevotella intermedia*, and *Treponema denticola* could produce substances such as hydrogen sulfide (H_2_S), and these have a certain toxicity and might lead to the occurrence of chronic inflammation in the gut (61). Additionally, some types of microbiota could also provoke a pro-inflammatory environment by activating the NF-kB pathway and increasing the expressions of pro-inflammatory cytokines (62, 63). Chronic inflammation has been verified to be associated with the development of CRC (64). Several studies have also found that bacterially produced genotoxins can induce CRC through DNA-damaging effects. *E. coli*, *Campylobacter jejuni*, and *Salmonella* have all been confirmed to induce CRC by producing specific genotoxins (65–67). During chronic inflammation, inflammatory cells in the gut might produce pro-oxidative molecules and reactive nitrogen species, and these substances could induce DNA damage or inactivate some relevant genes, thus causing tumorigenesis in the gut. Except for inflammatory cells, *E. faecalis*, *E. coli*, and *Bacteroides fragilis* could also directly or indirectly increase the levels of pro-oxidative molecules (68–70). The gut microbiota acts as an intermediary between diet and the host, and it plays an important role in host metabolism. On the one hand, different diet styles might cause varied microbiota that dominate in the gut: Diets with a high animal fat or protein content have been found to yield enterotypes abundant in *Bacteroides*, whereas diets with a high carbohydrate content have been found to yield enterotypes abundant in *Prevotella *(71, 72). On the other hand, the gut microbiota could produce detrimental or beneficial components *via* the metabolism of fats, proteins and fiber, and diets with a high fiber content increase the level of butyrate, which is a potent SCFA that prevents CRC (73). However, high-fat diets and high-protein diets promote tumorigenesis by increasing the concentrations of secondary fecal bile acid, H_2_S, and N-nitroso compounds (NOCs) in the gut, which are positively correlated with CRC (61, 74, 75). Overall, the human microbiota plays an important role in the development of CRC, but the relevant mechanisms are complex and have not yet been fully elucidated.

Our bibliometric analysis also plays a guiding role in the potential clinical applications of the human microbiome. As mentioned above, the human microbiome participates in the development and prevention of CRC *via* various mechanisms. Thus, human microbiota modulation that aims to restore normal human microbiota, especially gut flora, is a potential novel method to prevent and treat CRC. In recent years, multiple strategies, such as fecal microbiota transplantation (FMT), probiotics, prebiotics, postbiotics, and dietary interventions, have been proposed. FMT transfers stool transplants from donors to the gut of receivers through colonoscopy or oral administration. A former meta-analysis verified that FMT is an approved treatment method in resolving recurrent and refractory Clostridium difficile infection (CDI) (76). Although few studies have explored the clinical application of FMT in CRC, several animal experiments have revealed the potential therapeutic efficacy of FMT in treating CRC. Rosshart et al. (77) demonstrated that FMT (stool transplants from wild mice to laboratory mice) could prevent tumorigenesis in the gut caused by mutagen or inflammation agents. Some studies have also found that stool transplants from patients with CRC increase tumor formation in mice through different mechanisms of tumorigenesis (78, 79). Overall, FMT might be a promising method for the prevention and treatment of CRC in the future. Another intensively studied strategy is probiotics, which could interact with human gut flora and host cells to restore normal human microbiota, prevent tumorigenesis, and treat CRC. Some clinical studies have shown the efficacy of probiotics in inhibiting CRC development, improving the quality of life of patients with CRC and alleviating side effects of anticancer-related therapy (80–82). Additionally, some ingredients related to the human microbiota, such as prebiotics and postbiotics, have also been found to have a potential function in the treatment and prevention of CRC (83, 84). As previously described, high-fiber diets might prevent tumorigenesis by regulating the human microbiota; therefore, dietary intervention is regarded as another economical and rational method to prevent and treat CRC. A former systematic review of cohort studies revealed that a healthy diet habit that consists of vegetables, fruits, fish and poultry decreased the risk of CRC (85). Overall, restoring normal intestinal microbiota using various methods may prevent and treat CRC, and this might be another potential breakthrough in the management of CRC by regulating the human microbiome in the future.

Although the bibliometric analysis firstly provided insights into the current research status, hotspots, and trends in the field of CRC screening based on the microbiome, this study also has several limitations. First, we only included articles published in the English language, resulting in non-English publications not being analyzed, which may have interfered with the results of this bibliometric analysis. Second, we retrieved publications from the WoSCC database, and relevant publications published elsewhere, such as in PubMed, may not be found in the WoSCC database; this may also lead to some unpredictable biases. However, the WoSCC database is recognized worldwide and has a large number of multidisciplinary collections of published articles. Third, the short publication time of the recently published high-quality studies may have led to a low citation number. Thus, the citation frequency cannot truly reflect the quality of the publications.

## 5 Conclusions

Overall, we conducted a comprehensive review of publications related to CRC screening based on the human microbiome using a bibliometric analysis approach. A growing number of studies from multiple institutions and countries/regions have been published year by year, and the research in this field is becoming more in-depth and diversified. The current research hotspots mainly focus on the precancerous lesions of CRC that need to be screened, CRC screening *via* the gut-derived microbiome and the early detection of CRC. A future hotspot trend might be the combined analysis of microbiomics and metabolomics for CRC risk screening. The results of the current bibliometric analysis could serve as a valuable guide for relevant researchers to further understand the current research status and to grasp the potential future research direction in this field.

## Data availability statement

The original contributions presented in the study are included in the article/**Supplementary Material**. Further inquiries can be directed to the corresponding author.

## Author contributions

(I) Conception and design: JHZ, CL. (II) Administrative support: WD. (III) Provision of study materials or patients: FL, JXZ. (IV) Collection and assembly of data: JHZ, HX. (V) Data analysis and interpretation: CT, CL. (VI) Manuscript writing: All authors. All authors contributed to the article and approved the submitted version.
