# Supplementary material for: The global research of microbiota in colorectal cancer screening: a bibliometric and visualization analysis
Source: Front Oncol. 2023 May 5;13:1169369. doi: 10.3389/fonc.2023.1169369 (PMC10196493; doi:10.3389/fonc.2023.1169369)
Supplement: Supplementary file 1 [file Table_1.docx]

**Supplementary Material:**

Supplementary Table 1. Top 15 ranking table of authors, institutions, and countries based on WoSCC.

Supplementary Table 2. Top 10 ranking table of cited authors, cited journals, countries, and institutions based on CiteSpace.

Supplementary Table 1. Top 15 ranking table of authors, institutions, and countries based on WoSCC

| Rank | Authors | Number | Institutions | Number | Countries | Number |
| --- | --- | --- | --- | --- | --- | --- |
| 1 | Yu Jun | 14 | SHANGHAI JIAO TONG UNIVERSITY | 26 | CHINA | 214 |
| 2 | Fang Jing-Yuan | 10 | HARVARD UNIVERSITY | 23 | USA | 186 |
| 3 | Han ShuWen | 8 | HARVARD MEDICAL SCHOOL | 19 | ITALY | 45 |
| 4 | Li Huan | 8 | UNIVERSITY OF MICHIGAN | 18 | JAPAN | 40 |
| 5 | Ogino Shuji | 8 | UNIVERSITY OF MICHIGAN SYSTEM | 18 | ENGLAND | 37 |
| 6 | Sinha Rashmi | 8 | NATIONAL INSTITUTES OF HEALTH NIH USA | 17 | NETHERLANDS | 34 |
| 7 | Zhang J | 8 | CHINESE UNIVERSITY OF HONG KONG | 16 | FRANCE | 33 |
| 8 | Zhang L | 8 | FUDAN UNIVERSITY | 16 | GERMANY | 33 |
| 9 | Chan Andrew T | 7 | NIH NATIONAL CANCER INSTITUTE NCI | 15 | SPAIN | 29 |
| 10 | Li Y | 7 | CIBER CENTRO DE INVESTIGACION BIOMEDICA EN RED | 14 | SOUTH KOREA | 22 |
| 11 | Schloss Patrick D | 7 | INRAE | 14 | AUSTRALIA | 21 |
| 12 | Sears Cynthia L | 7 | UDICE FRENCH RESEARCH UNIVERSITIES | 14 | CANADA | 18 |
| 13 | Wang X | 7 | BRIGHAM WOMEN S HOSPITAL | 13 | IRAN | 17 |
| 14 | Yang J | 7 | CONSEJO SUPERIOR DE INVESTIGACIONES CIENTIFICAS CSIC | 13 | IRELAND | 15 |
| 15 | Yang Xi | 7 | HARVARD T H CHAN SCHOOL OF PUBLIC HEALTH | 13 | INDIA | 14 |

Supplementary Table 2. Top 10 ranking table of cited authors, cited journals, countries, and institutions based on CiteSpace

| Category | Rank | Cited Author | Number | Cited Journal | Number | Country | Number | Institution | Number |
| --- | --- | --- | --- | --- | --- | --- | --- | --- | --- |
| Frequency | 1 | KOSTIC AD | 183 | GUT | 441 | PEOPLES R CHINA | 207 | Shanghai Jiao Tong Univ | 23 |
|  | 2 | CASTELLARIN M | 118 | PLOS ONE | 418 | USA | 180 | Fudan Univ | 15 |
|  | 3 | RUBINSTEIN MR | 97 | GASTROENTEROLOGY | 375 | ITALY | 45 | Chinese Univ Hong Kong | 14 |
|  | 4 | ARTHUR JC | 95 | NATURE | 354 | JAPAN | 39 | Univ Michigan | 13 |
|  | 5 | SIEGEL RL | 89 | SCIENCE | 352 | ENGLAND | 35 | NCI | 12 |
|  | 6 | ZACKULAR JP | 84 | P NATL ACAD SCI USA | 292 | GERMANY | 33 | Brigham & Womens Hosp | 11 |
|  | 7 | ZELLER G | 82 | INT J CANCER | 272 | FRANCE | 33 | Harvard Med Sch | 11 |
|  | 8 | WONG SH | 78 | CELL HOST MICROBE | 259 | NETHERLANDS | 32 | Harvard TH Chan Sch Publ Hlth | 10 |
|  | 9 | MIMA K | 77 | CELL | 258 | SPAIN | 28 | German Canc Res Ctr | 9 |
|  | 10 | DEJEA CM | 75 | WORLD J GASTROENTERO | 252 | SOUTH KOREA | 21 | Sun Yat Sen Univ | 9 |
| Centrality | 1 | OHKUSA T | 0.2 | AM J CLIN NUTR | 0.14 | USA | 0.38 | NCI | 0.07 |
|  | 2 | SWIDSINSKI A | 0.14 | CANCER | 0.08 | ITALY | 0.15 | Sun Yat Sen Univ | 0.07 |
|  | 3 | BAYERDORFFER E | 0.12 | AM J PATHOL | 0.08 | FRANCE | 0.14 | German Canc Res Ctr | 0.05 |
|  | 4 | BARTHOLD SW | 0.12 | BIOCHEM BIOPH RES CO | 0.08 | DENMARK | 0.12 | Brigham & Womens Hosp | 0.04 |
|  | 5 | CUMMINGS JH | 0.11 | BRIT J CANCER | 0.06 | CZECH REPUBLIC | 0.09 | Harvard Med Sch | 0.04 |
|  | 6 | HAMILTON SR | 0.11 | CELL | 0.05 | INDIA | 0.08 | Helsinki Univ Hosp | 0.04 |
|  | 7 | ARTHUR JC | 0.1 | DIGEST DIS SCI | 0.05 | ARGENTINA | 0.07 | Harvard TH Chan Sch Publ Hlth | 0.04 |
|  | 8 | FEARON ER | 0.1 | BRIT J NUTR | 0.05 | SAUDI ARABIA | 0.07 | Shanghai Jiao Tong Univ | 0.04 |
|  | 9 | KOSTIC AD | 0.09 | AM J GASTROENTEROL | 0.05 | COLOMBIA | 0.06 | Guangzhou Med Univ | 0.03 |
|  | 10 | BARKER N | 0.09 | AM J CLIN PATHOL | 0.05 | GERMANY | 0.06 | Oslo Univ Hosp | 0.02 |
| Degree | 1 | KOSTIC AD | 80 | AM J CLIN NUTR | 127 | DENMARK | 30 | German Canc Res Ctr | 22 |
|  | 2 | SOBHANI I | 78 | CELL | 103 | USA | 29 | Brigham & Womens Hosp | 20 |
|  | 3 | ZACKULAR JP | 74 | GUT | 100 | CZECH REPUBLIC | 27 | NCI | 19 |
|  | 4 | ARTHUR JC | 72 | CANCER | 96 | COLOMBIA | 25 | Harvard Med Sch | 17 |
|  | 5 | RUBINSTEIN MR | 68 | GASTROENTEROLOGY | 94 | ARGENTINA | 24 | Helsinki Univ Hosp | 17 |
|  | 6 | MARCHESI JR | 64 | DIGEST DIS SCI | 90 | ITALY | 23 | Harvard TH Chan Sch Publ Hlth | 16 |
|  | 7 | ZELLER G | 62 | BRIT J CANCER | 87 | GREECE | 23 | Dana Farber Canc Inst | 16 |
|  | 8 | CASTELLARIN M | 61 | ALIMENT PHARM THER | 86 | BELGIUM | 22 | Shanghai Jiao Tong Univ | 15 |
|  | 9 | TJALSMA H | 59 | AM J PATHOL | 86 | AUSTRIA | 22 | Oslo Univ Hosp | 14 |
|  | 10 | WANG TT | 57 | NATURE | 83 | GERMANY | 21 | Sun Yat Sen Univ | 12 |
